# The Association of Serum Circulating Neuropeptide Q and Chemerin Levels with Cardiometabolic Risk Factors among Patients with Metabolic Syndrome

**DOI:** 10.3390/biom11121863

**Published:** 2021-12-10

**Authors:** Marta Pelczyńska, Aniceta Ada Mikulska, Krystyna Czyżewska, Paweł Bogdański, Teresa Grzelak

**Affiliations:** 1 Chair and Department of Treatment of Obesity, Metabolic Disorders and Clinical Dietetics, Poznan University of Medical Sciences, 84 Szamarzewskiego Street, 60-569 Poznań, Poland; pbogdanski@ump.edu.pl; 2 Chair and Department of Physical Pharmacy and Pharmacokinetics, Poznan University of Medical Sciences, 6 Święcickiego Street, 60-781 Poznań, Poland; amikulska@ump.edu.pl; 3 Department of Nursing, Stanislaw Staszic State University of Applied Sciences in Pila, 10 Podchorążych Street, 64-920 Piła, Poland; czyzew@ump.edu.pl; 4 Chair and Department of Physiology, Poznan University of Medical Sciences, 6 Święcickiego Street, 60-781 Poznań, Poland; tgrzelak@ump.edu.pl

**Keywords:** neuropeptide Q, chemerin, adipose tissue, obesity, diabetes, metabolic syndrome

## Abstract

The potential involvement of neuropeptide Q (NPQ) and chemerin (CHEM) in metabolic disorders is yet to be fully elucidated. The aim of this study was to evaluate serum concentrations of NPQ and CHEM and to establish their relationship with cardiometabolic risk factors among individuals with metabolic syndrome. A total of 66 patients with metabolic syndrome (MetS) and 83 healthy volunteers (non-MetS) underwent biochemical, blood pressure, and anthropometric measurements. The concentration of NPQ in the MetS group was significantly lower (0.47 (0.34 ; 0.54) vs. 0.52 (0.43 ; 0.60) ng/mL, *p* = 0.015) than in non-MetS, while there were no differences in CHEM level. In the entire study population, we observed several negative correlations between NPQ concentration and waist-hip ratio (WHR), visceral adipose tissue, diastolic blood pressure (DBP), triglycerides (TG) along with a positive correlation with high-density lipoprotein (HDL), total muscle mass, and CHEM. Moreover, a negative correlation was observed in the MetS group between NPQ and glycemia. CHEM showed no significant correlations with cardiometabolic risk factors in the study population. In a multiple regression model, the total muscle mass proved to be an independent factor determining NPQ concentration in the population (*p* < 0.00000001, *R*^2^*_adj_* = 28.6%). NPQ seems to protect against metabolic disorders correlated with obesity. Thus, it is worth considering NPQ level as a candidate protective biomarker of metabolic syndrome complications.

## 1. Introduction

Neuropeptides and adipokines, which are secreted mainly by adipose tissue and other organs, are involved in the pathogenesis of metabolic syndrome (MS). Neuropeptide Q (NPQ), also known as a spexin, is a newly discovered peptide hormone. Identified in 2007, NPQ is a product of the Ch12orf39 gene, consisting of fourteen amino acid residues [1]. Chemerin (CHEM), encoded by the retinoic acid receptor responder 2 gene (RARRES2), also known as tazarotene-induced gene 2 (TIG2), is an adipokine secreted by adipose tissue and other cells such as hepatocytes, small intestine, or kidneys [2]. Although neuropeptide Q and chemerin are widely expressed in endocrine and epithelial tissue, their involvement in human physiological functions is yet to be fully established.

The natural ligand for NPQ is a galanin receptor 2/3 (GALR2/3) [3]. Neuropeptide Q and galanin induces a specific active conformation of GALR2, activating different signals via the same receptor [4]. As a neuropeptide, Galanin regulates numerous physiological functions including feeding and energy homeostasis. NPQ may thus be assigned a role in the regulation of food intake and body weight, as well as lipid and carbohydrate metabolism. Moreover, it has been suggested that NPQ is involved in controlling the proliferation of adrenocortical cells or the functionating of the cardiovascular, renal, and endocrine system. NPQ is associated with a number of different disorders, including obesity, type-2 diabetes, fatty liver disease, and mental disabilities such as anxiety and depression [5].

Chemerin is a chemokine, secreted as an inactive propeptide (preprochemerin 18 kDa). The active form of this protein (16 kDa) is produced by cleaving the C-terminal amino acids [6]. CHEM has been shown to participate in the pathogenesis of a variety of proinflammatory and metabolic diseases, including obesity, diabetes, and cardiovascular disease. Nevertheless, the biological function of chemerin as a proinflammatory or anti-inflammatory modulator remains controversial. On one hand, CHEM enhances the chemotaxis of immature dendritic cells and macrophages by activating the CMKLR1 and CCRL2 receptors, bridging the innate and adaptive immunity for the initiation of immune response [7,8]. On the other hand, chemerin reduces the number of immune cells at inflammatory sites, and thus the expression of proinflammatory cytokines [9]. These findings suggest that CHEM may indicate both pro- and anti-inflammatory processes, depending on the biological system. It is worth noting that chemerin is mainly expressed in visceral adipose tissue. By acting on CMKLR1 receptors, it affects inflammation, angiogenesis, and adipogenesis in cells of adipose tissues. This chemokine thus participates in the regulation of lipid and glucose metabolism [10].

Elevated waist circumference correlated with obesity is a component of metabolic syndrome, alongside hypertension, dyslipidemia, and hyperglycemia. The prevalence of excessive body weight has emerged as a serious global issue, reaching a pandemic level. According to the World Health Organization (WHO), more than 1.9 billion people are overweight, of whom about 600 million are classified as obese. The prevalence of excessive body weight has doubled since 1980, and in many European countries, it has even tripled [11]. The pathogenesis of the metabolic syndrome has not been fully explained. Several factors predisposing to the manifestation of MS have been described, including genetic and environmental factors such as a high-caloric atherogenic diet and low physical activity [12]. The dominant traits of metabolic syndrome involve excessive amounts of visceral adipose tissue and insulin resistance. The dysfunction of adipose tissue in obese subjects predisposes them to secrete a wide variety of molecules as adipokines and neuropeptides [13]. Some of these, such as chemerin, contribute to so-called low-grade inflammation, creating a cluster of metabolic aberrations [14,15], while others such as NPQ seem to protect from those disorders [16]. Thus, it currently seems that neuropeptides and adipokines such as neuropeptide Q and chemerin play and important role in the development of metabolic disorders associated with obesity. For this reason, we undertook this study.

The aim of our study was to evaluate serum concentrations of neuropeptide Q and chemerin and to establish their relationship with cardiometabolic risk factors in individuals with metabolic syndrome.

## 2. Materials and Methods

### 2.1. Study Population

A total of 149 Caucasian individuals aged 24–66 years were examined (117 women and 32 men). As the study was voluntary, and women more often expressed their willingness to participate in analysis involving inter alia (i.a.) anthropometric measurements, is what might have caused the gender differences. Patients were recruited at the Department of Internal Medicine, Metabolic Disorders and Hypertension at Poznań University of Medical Sciences. Recruitment for both groups was conducted simultaneously over 24 months. Subjects were divided into two groups: 66 patients with metabolic syndrome (MetS, study group, 45.33 ± 9.59 years) and 83 volunteers without MetS (non-MetS, control group, 41.29 ± 8.19 years). The groups enrolled patients of similar age.

All participants were screened for MS according to the criteria of the International Diabetes Federation, the American Heart Association, and the National Heart, Lung, and Blood Institute from 2009. MS was diagnosed based on the presence of at least three of the five components of this syndrome, which included a waist circumference greater than 80 cm for women and 94 cm for men, an increased level of fasting blood glucose (FBG > 5.6 mmol/L), increased arterial blood pressure (>130 mmHg for systolic blood pressure—SBP, or >85 mmHg for diastolic blood pressure—DBP), hypertriglyceridemia (>1.7 mmol/L), and lowered concentration of high-density lipoprotein (HDL < 1.3 mmol/L for women and <1.0 mmol/L for men), or adequate drug therapy for hyperglycemia, hypertonia, arterialis, or dyslipidemia [17].

The exclusion criteria for both groups were pregnancy, lactation, secondary obesity or secondary hypertension, a history of cancer over the last five years, chronic heart or liver failure, autoimmune diseases, alcoholism, acute symptoms of infection in the three months prior to the study, ischemic and heart diseases, as well as liver, blood, hepatic, renal, adrenal, and thyroid disorders. The inclusion criteria were an age of 21 to 65 and the presence or absence of metabolic syndrome in the study and control group, respectively.

The study was approved by the Local Bioethics Committee (regulation no. 729/17 (approval date 22 June 2017) and 326/18 (approval date 8 March 2018)). Participation in the study was voluntary. Each participant provided written informed consent after having been informed about the project’s purpose and course. The study was conducted in accordance with the Declaration of Helsinki [18].

### 2.2. Study Design

#### 2.2.1. Anthropometric Parameters

After qualifying the patient for examination, a detailed history of diagnoses and medications was collected. The anthropometric measurements were taken after an overnight fasting of at least 12 h. Patients were dressed only in light underwear, with no shoes, during anthropometric measurements. Body weight and height were measured with accuracy to 0.1 kg (using a certified weighing scale) and to 0.1 cm (stadiometer). Waist and hip circumferences were determined using standard medical instruments. Waist circumference was measured at the midway between the costal arch and the upper iliac crest, and hip circumference at the level of the greater trochanters. Height, waist, and hip circumference were determined to an accuracy of 0.5 cm. These measurements were used to calculate the body mass index (BMI), calculated as body weight divided by height squared (kg/m^2^), and the waist-hip ratio (WHR), calculated as waist measurement divided by hip measurement. The Muscle Mass Index was calculated as muscle mass weight divided by height squared (kg/m^2^).

Body composition measurements were made using bioelectrical impedance analysis with a TANITA BC-418 device (Tanita, Tokyo, Japan). This was used to estimate body adipose tissue and lean body mass, i.e., total muscle mass and water content. Patients were advised to avoid consuming large amounts of fluid before the test and to discontinue intense physical exercise 12 h before the measurement.

#### 2.2.2. Blood Pressure Measurement

Blood pressure was measured manually three times in the morning using a sphygmomanometer after a ten-minute sitting rest, in accordance with the standard guidelines of the European Society of Hypertension (ESH) and the European Society of Cardiology (ESC) [19]. Three measurements were taken from each person in a sitting position over a two-minute interval. A properly selected cuff was used (standard 13 cm wide and 35 cm long, and wider for people with an arm circumference > 32 cm). The cuff was placed at the height of the heart. Based on three measurements, the mean systolic and diastolic blood pressure values were calculated. Following the ESC/ESH recommendations, hypertension was diagnosed as office SBP values of at least 140 mmHg or DBP values of at least 90 mmHg [19].

#### 2.2.3. Biochemical Measurements and Calculations

Blood samples of about 5 mL were collected from all study participants after overnight fasting (≥ 12 h after the last meal) and an all-night rest between 7:00 and 8:00 in the morning. Most of the serum samples were subjected to biochemical analysis immediately after collection (glycemia and lipid profile), whereas some of the other samples were separated, secured, and frozen at −80 °C until analysis could be performed. Fasting blood glucose, total cholesterol (TC), high-density lipoprotein, and triglycerides (TG) were determined using the enzymatic method with standardized commercial tests (Cobas c, Roche Diagnostic, Mannheim, Germany). The low-density lipoprotein (LDL) concentration was calculated as LDL = TC − (HDL + TG/2.2) [20]. To exclude external factors (fat- and carbohydrate-rich meals in the previous days or strenuous exercise) affecting biochemical measurements, all participants had a pedometer, and their diet was evaluated by a nutritional questionnaire.

#### 2.2.4. Neuropeptide Q and Chemerin Measurement

Neuropeptide Q serum concentrations were analyzed using commercial immunoenzymatic tests purchased from Phoenix Pharmaceuticals (Burlingame, CA, USA) with intra-assay and inter-assay variations below 10% and 15%, respectively. Chemerin concentration was also determined with a Mediagnost enzyme immunoassay kit (Reutlingen, Germany) with intra-assay and inter-assay variations below 2.17% and 5.16%, respectively. The assays were conducted in line with the manufacturer’s instructions. An enzyme-substrate reaction was provoked with a substrate for chromogen, and the color was monitored on an ELISA MR-96 microplate reader manufactured by Clindiag Systems (Pollare, Belgium). The concentration of NPQ and CHEM were calculated based on calibration curves, which were determined using a four-parameter algorithm (SigmaPlot 11.00, Systat Software, San Jose, CA, USA), prepared each time for the specific set of assays.

#### 2.2.5. Statistical Analysis

Statistical analysis of the results was conducted using Statistica 13 software with Medical Set (StatSoft, Tulsa, OK, USA). The results are presented as means ± standard deviations (SDs), or as medians with upper and lower quartiles. The results were analyzed statistically, using elements of descriptive statistics and statistical procedures, such as correlation analysis (Pearson’s for parametric distributions and Spearman’s for nonparametric distributions). Comparisons between groups were performed using the Mann–Whitney U test, or the unpaired Student’s *t*-test where the data were normally distributed. Stepwise multiple regression analysis was used to determine the predictor for NPQ and CHEM levels in blood. The level of statistical significance was taken as *p* < 0.05.

## 3. Results

### 3.1. Levels of Analysed Parameters

A total of 149 people aged 43.08 ± 9.03 years were enrolled into the study. The patients from the study group (MetS) presented, respectively, three (54.6%), four (22.7%), or five (22.7%) components of metabolic syndrome. Subjects from MetS group had statistically higher body weight (by 47.3%; *p* < 0.001), BMI (by 43.2%; *p* < 0.001) and percentage of body fat mass (by 38.3%; *p* < 0.001), as well as lower muscle mass (by 30.1%; *p* = 0.0001) and Muscle Mass Index (by 39%; *p* = 0.0001), than did subjects from the control group (non-MetS; Table 1). In addition, according to the definition of metabolic syndrome, the study group had significantly higher levels of FBG (by 8.9%; *p* < 0.001) and TG (by 112.2%; *p* < 0.001), and significantly lower concentrations of HDL (by 33.3%; *p* = 0.006), than the control group. The medians (first; third quartile) of concentrations of TC and LDL were slightly higher in the MetS group (5.03 (4.55 ; 5.87) mmol/L for TC and 3.06 (2.76 ; 3.94) mmol/L for LDL) than in the non-MetS group (4.97 (4.34 ; 5.79) mmol/L for TC and 3.15 (2.50 ; 3.70) mmol/L for LDL), although the difference was not statistically significant (*p* > 0.05). The mean systolic and diastolic blood pressures were higher in the metabolic syndrome patients (by 18.6% and 21.6% for SBP and DBP, respectively; *p* < 0.001) than in the control. The blood pressure, anthropometric, and biochemical characteristics of the MetS and non-MetS groups are summarized in Table 1.

Neuropeptide Q serum concentration in metabolic syndrome subjects was significantly lower than in the controls (0.47 (0.34 ; 0.54) vs. 0.52 (0.43 ; 0.60) ng/mL; *p* = 0.015). Surprisingly, no differences were noted in the serum chemerin concentration between MetS and non-MetS group (Table 1). It is worth adding that the grouping by body mass index size resulted in an increase in the median value of CHEM concentration in people with overweight and obesity (*n* = 108), compared to normal-weight subjects (*n* = 41) as 55.13 (38.32 ; 94.57) ng/mL for BMI > 25 kg/m^2^ and 53.06 (42.90 ; 66.85) ng/mL for BMI < 24.9 kg/m^2^; this difference also lacked statistical significance (*p* > 0.05).

### 3.2. Correlation between NPQ, CHEM, and Anthropometric and Biochemical Parameters

NPQ was positively correlated in the entire population with total muscle mass (*R* = 0.498, *p* < 0.0001), muscle mass index (MMI; *R* = 0.486, *p* < 0.0001), HDL (*R* = 0.211, *p* = 0.01), and CHEM (*R* = 0.217, *p* = 0.009) (Table 2). Moreover, NPQ was correlated negatively with waist–hip ratio (WHR; R = −0.195, *p* = 0.017), visceral adipose tissue (*R* = −0.248, *p* = 0.003), DBP (*R* = −0.197, *p* = 0.016), and TG (*R* = −0.176, *p* = 0.032). In the MetS group, NPQ was correlated positively with total muscle mass (*R* = 0.398, *p* = 0.002), MMI (*R* = 0.406, *p* = 0.001) and negatively with FBG (*R* = −0.245, *p* = 0.049). In the subjects without metabolic syndrome, a positive correlation was seen again in case of NPQ with total muscle mass (*R* = 0.545, *p* < 0.0001), MMI (*R* = 0.521, *p* < 0.001), and CHEM (*R* = 0.235, *p* = 0.033).

In the entire group, CHEM was positively correlated with total muscle mass (*R* = 0.276, *p* = 0.001) and MMI (*R* = 0.286, *p* < 0.001). The metabolic syndrome patients showed no relationship between concentrations of CHEM and biochemical or anthropometric parameters. In the group without metabolic syndrome, CHEM correlated positively only with MMI (*R* = 0.346, *p* = 0.001).

### 3.3. Regression Model

In the multiple regression model including four variables (sex, chemerin, HDL level, and total muscle mass in kg), sex and the total muscle mass proved to be independent factors that determine the concentration of serum NPQ in the entire examined population (*p* < 0.00000001, *R*^2^*_adj_* (adjusted R-squared) = 28.6%, Table 3).

## 4. Discussion

This is the first study to show that total muscle mass is an independent determinant of serum neuropeptide Q concentration in obese individuals with metabolic syndrome. The serum concentration of NPQ was significantly lower in MetS group while there was surprisingly no difference in CHEM level between the groups. Several negative correlations between serum NPQ concentration and cardiometabolic risk-related parameters, along with a positive HDL correlation, were observed in the entire studied population.

The study of Walewski et al. demonstrated that NPQ gene expression was downregulated by a factor of 14.9 in the omental and subcutaneous adipose tissue of obese subjects, and was negatively correlated with leptin level. The same study showed that, in diet-induced obese (DIO) mice/rats, NPQ administration resulted in food intake limitation and body weight reduction [21]. In line with our study, Kolodziejski et al. in their research confirmed that serum neuropeptide Q level was lower in women with excessive body weight and correlated negatively with BMI and insulin concentration [22]. Karaca et al. also indicated a decreased NPQ level in patients with type 1 and 2 diabetes [23]. On the other hand, some analyses did not confirm these reports: Hodges et al. declined the proposed role of NPQ as a biomarker of metabolic processes in adolescents, finding no correlations between its concentration and body composition or biochemical parameters [24]. In another study in pregnant women, NPQ was not associated with gestational diabetes mellitus or obesity [25].

In our entire whole study population, NPQ was negatively correlated with cardiometabolic risk-related parameters, such as WHR, percentage of adipose tissue, DBP, and TG. Our results are confirmed by other studies that determined reduced NPQ level in type 2 diabetes patients, as well as a negative correlation between it and glycemia and lipids parameters, including with triglyceride concentrations [26]. Lin et al., in a study conducted in 2018 in healthy adult women, showed that the circulating neuropeptide Q level was negatively correlated with BMI, FBG, TG level, and age, suggesting a possible role of this peptide other than in aging-related dysfunctions [27], while also indicating its potential role in energy, glucose, and lipid metabolism [5]. Several mechanisms have been proposed in this regard, including the NPQ effects reducing food intake and increasing energy expenditure, as well as changes in the respiratory exchange ratio (promoting the burning of adipose tissue in preference to carbohydrate) [28]. It is worth mentioning that, in one animal study, administration of NPQ in dietary-induced obese mice inhibited the uptake of long chain fatty acids (LCFA) into adipocytes; thus, it is speculated that the absence of this molecule may be critical to hormonal regulation in obese subjects [21].

Our study showed a negative correlation between NPQ and fasting glucose level in patients with metabolic syndrome. Other studies have confirmed this correlation [25,26,27]. Studies of NPQ have shown a decrease in insulin secretion under hyperglycemic conditions (suggesting an inhibition of insulin resistance and an increase in insulin sensitivity) in an analysis performed on rat pancreatic cells and the INS-1 cell line. In the latter model, there was also a decrease in the expression of the gene for insulin and the PDX (insulin promoter factor) gene, and an increase in cell survival and proliferation [29]. Moreover, colocalization of β-cells and NPQ has been shown in humans, suggesting that this molecule may affect pancreatic function [26,29]. As previously mentioned, the natural ligand for NPQ is GALR2/3 [3]. Neuropeptide Q and galanin thus activate specific conformations of GALR2 and induce different signals via the same receptor [4]. In a previous study, a positive correlation was described between galanin and metabolic disorders, including fasting blood glucose (FGB), glycated hemoglobin (HbA1c), and TG levels [30,31]. Some animal and human studies showed that galanin improves insulin sensitivity and glucose clearance by enhancing glucose transporter type 4 receptors and suppressing glucose-stimulated insulin secretion [32,33]. These findings suggest that neuropeptide Q may act via the same mechanisms as galanin. Nevertheless, in future studies, it would be worth determining other parameter characterizing insulin resistance (such as fasting insulin and HOMA-IR index) to evaluate the specific role of NPQ in carbohydrate dysfunctions.

We showed for the first time that, in a multiple regression model, total muscle mass is an independent determinant of serum neuropeptide Q concentration. Moreover, in the entire analysis, we found a significant positive correlation between NPQ level and total muscle mass, as well as MMI value. The two largest organs in the human body are skeletal muscle system and the adipose tissue. The first is mainly an effector organ and the second one performs energy storage. Furthermore, both have an endocrine function, secreting different cytokines. In many locations, adipose tissue is adjacent to muscle, so they can both induce multiple signaling pathway affecting metabolic processes [15]. Although Gu et al. have shown low NPQ gene expression in the skeletal muscle (similar to our results), its level was reduced in type 2 diabetes mellitus (T2DM) patients and correlated negatively with FGB, HbA1c, TG, and LDL [26]. In another study, plasma NPQ level was decreased in obese participants with or without T2DM, compared to normal-weight subjects, and correlated negatively with adiposity markers. It is worth noting that, in both obese groups, the NPQ level significantly increased in response to regular physical exercise, in parallel with increased oxygen consumption (VO_2_ max) and with an improvement in metabolic profile [34]. It thus seems that the total content of muscle mass in the human body may determine a total serum concentration of NPQ, as well as its correlation with the occurrence of metabolic disorders. In obese subjects, the reduction in total muscle mass and the extension of body adipose tissue may predispose to a decrease in the concentration of NPQ. The main goal of therapy for patients with metabolic syndrome should be lifestyle modification, including physical activity alongside dietary interventions [35,36]; dietary interventions lead to both the reduction of adipose tissue and muscle mass normalization. According to the recommendation of WHO, people with chronic conditions (including diabetes and hypertension) should perform at least 150–300 min of moderate-intensity aerobic physical activity, or at least 75–150 min of vigorous-intensity aerobic physical activity per week (or a combination of these exercises) to improve muscular, bone, and functional health [37].

Surprisingly, our study did not show any differences in chemerin concentration between the groups. Circulating CHEM level is usually higher in the patients with obesity, diabetes, and cardiovascular disease—that is, in those with metabolic syndrome [38,39]. It is involved in metabolism throughout the whole body, and particularly in energy expenditure and insulin sensitivity. Some studies have shown a positive correlation between CHEM circulating levels and BMI and obesity-related biomarkers, such as low-grade inflammation, blood pressure, and insulin resistance [40,41]. On the other hand, and in line with our results, not all studies have confirmed this relationship, so the association of serum chemerin levels with metabolic syndrome cannot be considered to have been clearly established [14,42].

The effects of CHEM on the development of insulin resistance in individuals with metabolic syndrome is controversial. On one hand, it has been shown that increased expression of the chemerin gene in the 3T3-L1 cell line resulted in increased insulin-induced phosphorylation of the insulin receptor substrate 1 (IRS1) tyrosine residue and higher glucose uptake [43]. In contrast, Kralisch et al. showed an inverse relationship in the same cell line. In their study, CHEM significantly reduced insulin-stimulated glucose uptake [44]. The varying results for the concentration and importance of CHEM may be due to anti-inflammatory and proinflammatory effects of its various derivatives, which have the ability to bind to the same receptor. Depending on local conditions (such as the presence of carboxypeptidase B2, carboxypeptidase N, cathepsin G, cathepsin K, leukocyte elastase, and fibrinolytic enzymes such as plasmin), different amino acids may separate from prochemerin, leading to the formation of short-chain peptides with different effects. In our study, we measured total CHEM concentrations in the blood, which may explain the lack of difference in levels between the groups with and without metabolic syndrome [9,45]. Moreover, we showed a positive association between NPQ and CHEM level. To date no other studies have analyzed the concentration of these two molecules. We suspect that this correlation may be the effect of the protective action of NPQ in terms of metabolic disorders intensified by chemerin.

Our study has some limitations. To ensure a homogeneous study population, the study groups were limited by numerous exclusion criteria. The study excluded people over 66 years of age due to decreases in their total muscle mass and basic metabolism rate. Moreover, the study contained only Caucasian people. In further studies, it is worth considering expanding the study population, including more diverse populations and larger research groups, especially men. It would also be valuable to evaluate biochemical parameters (especially fasting glucose and triglyceride levels) under different conditions as test meal or glucose-loaded test. The most effective ways of analyzing body composition are magnetic resonance imaging or DEXA method (the last one characteristic: very low radiation exposure). Our study instead used the electrical bioimpedance method, along with anthropometric measurements. Nevertheless, above mentioned methods are widely accepted in clinical trials. Furthermore, factors that might affect NPQ and CHEM concentration, such as dietary intake, physical activity, and drugs were not taken into consideration.

The greatest strengths of our study include its in-depth comprehensive analysis of biochemical, physiological, and anthropometric parameters in patients with metabolic syndrome. Another strong point is the exclusion of patients with secondary forms of obesity. Furthermore, to the best of our knowledge, this is the first study to identify that total muscle mass is an independent determinant of serum neuropeptide Q concentration in individuals with metabolic syndrome. Our research thus provides an insight into the cardiometabolic risk factors correlated with obesity.

## 5. Conclusions

Patients with metabolic syndrome present lower serum neuropeptide Q concentrations than subjects without metabolic disorders but surprisingly no differences were seen in the chemerin level between these groups. Several negative correlations between serum NPQ concentration and cardiometabolic risk-related parameters were found, as was a positive correlation with HDL. Moreover, total muscle mass was found to be an independent determinant of serum NPQ concentration in individuals with metabolic syndrome. NPQ thus seems to protect against metabolic disorders correlated with obesity. Given our results, it is worth considering neuropeptide Q serum concentration as a candidate protective biomarker of metabolic syndrome complications. Nevertheless, further studies on this issue are required.

## Figures and Tables

**Table 1:** Blood pressure and anthropometric and biochemical characteristics of participants, by presence of metabolic syndrome.

Parameter [unit]	MetS (*n* = 66)	non-MetS (*n* = 83)	*p*-Value
SBP [mmHg]	140.00 (135.25 ; 150.00)	118.00 (107.50 ; 129.00)	*p* < 0.001 **
DBP [mmHg]	90.00 (80.50 ; 100.00)	74.00 (68.00 ; 82.00)	*p* < 0.001 **
Body weight [kg]	105.05 (86.55 ; 125.80)	71.30 (62.05 ; 79.70)	*p* < 0.001 **
BMI [kg/m^2^]	35.80 (31.15 ; 43.18)	25.00 (21.55 ; 28.45)	*p* < 0.001 **
WHR	0.95 ± 0.09	0.81 ± 0.06	*p* < 0.001 *
Total body fat mass [%]	42.72 ± 8.23	30.90 ± 7.26	*p* < 0.001 *
Total muscle mass [kg]	30.10 (23.30 ; 46.15)	43.10 (36.70 ; 46.90)	0.0001 **
MMI [kg/m^2^]	9.41 (8.41 ; 16.46)	15.43 (12.28 ; 16.76)	0.0001 **
FBG [mmol/L]	5.51 (5.21 ; 6.18)	5.06 (4.70 ; 5.22)	*p* < 0.001 **
TC [mmol/L]	5.03 (4.55 ; 5.87)	4.97 (4.34 ; 5.79)	0.634 **
TG [mmol/L]	2.08 ± 1.13	0.98 ± 0.44	*p* < 0.001 *
HDL [mmol/L]	1.18 ± 0.30	1.77 ± 0.42	0.006 *
LDL [mmol/L]	3.06 (2.76 ; 3.94)	3.15 (2.50 ; 3.70)	0.475 **
NPQ [ng/mL]	0.47 (0.34 ; 0.54)	0.52 (0.43 ; 0.60)	0.015 **
CHEM [ng/mL]	51.57 (38.99 ; 80.95)	55.35 (40.41 ; 94.59)	0.368 **

Parameters are shown as means (± standard deviations) or medians (first; third quartile); MetS: study group; non-MetS: control group; *n*: number of participants; *p*-value: level of statistical significance; SBP: systolic blood pressure; DBP: diastolic blood pressure; BMI: body mass index; WHR: waist–hip ratio; MMI: muscle mass index; FBG: fasting blood glucose; TC: total cholesterol; TG: triglycerides; HDL: high-density lipoprotein; LDL: low-density lipoprotein; NPQ: neuropeptide Q; CHEM: chemerin; * level of statistical significance for STUDY vs. CONTROL groups according to Student’s *t*-test; ** level of statistical significance for STUDY vs. CONTROL groups according to the Mann–Whitney U test.

**Table 2:** Indices of correlation and levels of statistical significance for the relationship between NPQ and selected anthropometric and biochemical parameters in the entire population (*n* = 149).

Variables [Unit]	Correlation Coefficient (*R*)	*p*-Value *
WHR	−0.195	0.017
Visceral adipose tissue [%]	−0.248	0.003
Total muscle mass [kg]	0.498	<0.0001
MMI [kg/m^2^]	0.486	<0.0001
DBP [mmHg]	−0.197	0.016
HDL [mmol/L]	0.211	0.01
TG [mmol/L]	−0.176	0.032
CHEM [ng/mL]	0.217	0.009

*p*-value: statistical significance; NPQ: neuropeptide Q; WHR: waist–hip ratio; MMI: muscle mass index; DBP: diastolic blood pressure; HDL: high-density lipoprotein; TG: triglycerides; CHEM: chemerin; *n*: number of participants; Spearman rank correlation test *.

**Table 3:** Comparison of the stepwise multiple regression model explaining variations in NPQ levels before and after adding the fourth variable (total muscle mass) to the basic model, which includes sex, chemerin, and HDL levels in the entire examined population (*n* = 149).

Statistical Parameters	Model with Two Variables (Sex, Chemerin, and HDL Level)	Extended Model Incorporating Total Muscle Mass as New Variable
*R* ^2^ *_adj_*	0.139	0.286
*F*	23.006	28.238
*p*-value	0.000004	<0.00000001

*p*-value: statistical significance; *n*: number of participants; NPQ: neuropeptide Q; HDL: high-density lipoprotein; *R*^2^*_adj_:* adjusted R-squared; *F*: F statistic; *p*: level of statistical significance in multiple regression.

## Data Availability

The data presented in this study are available on request from the corresponding author. The data are not publicly available due to privacy restrictions.
